# Spenectomy in Pernicious Anaemia

**Published:** 1915-03

**Authors:** 


**Spenectomy in Pernicious Anaemia.** Herbert C. Moffit, "American Journal of the Medical Science," December, 1914, from a study of 33 cases, including 2 personally observed in detail, deduces the following: immediate mortality 8 cases, 25%; in most cases, large numbers of megaloblasts, normoblasts and Howell-Jolly bodies appeared soon after operative recovery. Symptomatic improvement usually followed, sometimes beyond that indicated by the blood. The operation does not, however, seem to produce permanent results or results differing essentially from spontaneous ameliorations that are well recognized.

